# Lifetime Cannabis Use Is Not Associated With Negative Beliefs About Medication in Patients With First Treatment Psychosis

**DOI:** 10.3389/fpsyt.2022.824051

**Published:** 2022-03-29

**Authors:** Priyanthi B. Gjerde, Synne W. Steen, Trude S. J. Vedal, Nils Eiel Steen, Elina J. Reponen, Ole A. Andreassen, Vidar M. Steen, Ingrid Melle

**Affiliations:** ^1^Norwegian Centre for Mental Disorders Research, Department of Clinical Science, University of Bergen, Bergen, Norway; ^2^Dr. Einar Martens Research Group for Biological Psychiatry, Department of Medical Genetics, Haukeland University Hospital, Bergen, Norway; ^3^Research Unit for General Practice, NORCE Norwegian Research Centre, Bergen, Norway; ^4^Division of Mental Health, Akershus University Hospital, Oslo, Norway; ^5^NORMENT, Oslo University Hospital, Oslo, Norway; ^6^Institute of Clinical Medicine, University of Oslo, Oslo, Norway

**Keywords:** psychosis, schizophrenia, cannabis, substance abuse, BMQ

## Abstract

**Objective:**

Cannabis use is common among patients with psychosis, and along with negative beliefs about medication, it has been found to predict poor adherence to antipsychotic drug treatment. Such lack of adherence to antipsychotic drug treatment increases the risk of poor clinical outcomes and relapse in patients with first treatment for psychosis (FTP). However, to date, it is unclear whether cannabis use may be related to negative perceptions about antipsychotic drug treatment.

**Methods:**

A cross-sectional sample of 265 FTP patients with schizophrenia spectrum disorder underwent extensive clinical assessments. Three measures of cannabis use were obtained: lifetime, current and meeting diagnostic criteria for abuse or addiction. For the primary analyses we focused on lifetime cannabis use. The Beliefs about Medication Questionnaire (BMQ) was employed to assess the patients' specific concerns and perceptions of antipsychotic medications, as well as general beliefs about pharmacotherapy. The relationship between lifetime cannabis use and BMQ scores was investigated with general linear model (GLM) analyses, controlling for age and sex.

**Results:**

Patients with lifetime use of cannabis ≥10 times were more likely to be male, younger at the age of onset of psychosis and with higher levels of alcohol use and daily tobacco smoking, as compared to the non-users (*p* < 0.05). Neither lifetime use of cannabis, current use nor a cannabis abuse diagnosis was associated with negative beliefs about medicines as measured by the BMQ questionnaire.

**Conclusion:**

Use of cannabis is not linked to negative perceptions about antipsychotic medicines in patients with FTP. Other reasons for poor compliance to antipsychotic drug treatment in cannabis users need to be further investigated.

## Introduction

People with schizophrenia have high comorbidity of substance use disorders (1), in particular cannabis use disorder (2, 3). In patients coming to their first treatment for psychosis (FTP), lifetime exposure to cannabis has been estimated to be up to 80% and current use in at-risk subjects estimated to be 30–40% (4, 5). Cannabis use has been associated with an earlier onset of psychosis, more severe course of the illness, stronger impairment of global functioning, and a higher risk of relapse (4, 6–11). While some authors interpret the increased cannabis use in schizophrenia as a means of self-medication to alleviate psychotic symptoms (12), recent findings indicate that cannabis use also may be a causal factor in developing schizophrenia (13–17) predating the onset of prodromal symptoms (13, 18–20).

Antipsychotic drugs are central to the treatment of severe mental disorders, but it is often difficult to encourage patients to stay on these medications over time. Up to two-thirds of patients with schizophrenia comply poorly to prescribed antipsychotic treatments (21), with increased risks of relapse, hospitalization, and suicide (22). Cannabis use, poor insight and negative beliefs about medication have been found to be significant predictors of poorer compliance in this patient group (23–27). In a previous study of FTP patients, a link between negative attitude and beliefs about medication and adherence to antipsychotic drug treatment was demonstrated (28). Given that cannabis use also has been found to predict poor compliance to antipsychotic drug treatment (23), it is of interest to explore if cannabis use is associated with negative beliefs about medicines. However, to date, no such relationship has been systematically explored. This question is particularly relevant to study in FTP patients because cannabis use may be a modifiable risk factor for treatment non-adherence, while negative attitudes toward medication may additionally decrease antipsychotic drug adherence with consequences for both the patient (e.g., illness course and outcome) and the society (e.g., readmissions and longer hospital stays).

Negative opinions and perceptions about medication can be measured using a self-reporting form called Beliefs about Medicines Questionnaire (BMQ) (29), which has been shown useful for patients with severe psychiatric conditions including schizophrenia (28). The primary purpose of this study was to investigate whether cannabis use is associated with negative attitudes toward antipsychotic drugs, as well as medicines in general, in FTP with schizophrenia spectrum disorders. Based on previous literature of cannabis use and negative believes about medication being related to poorer drug compliance, we hypothesized that cannabis use would be linked to negative perceptions of antipsychotic drug treatment.

## Methods

### Study Design and Patients

The present cross-sectional study, which was part of the larger Thematically Organized Psychosis (TOP) study, Oslo, Norway, included 265 patients with schizophrenia spectrum disorder who had started their first treatment within the last 12 months. Details regarding recruitment to the TOP-study are described elsewhere (30). In short, the inclusion criteria were (1) age 18–65 years, (2) meeting criteria for a broad schizophrenia spectrum psychosis diagnosis according to the Diagnostic and Structural Manual of Mental Disorders, fourth version [DSM-IV, (31)] (i.e., schizophrenia, schizophreniform disorder, schizoaffective disorder, delusional disorder, brief psychotic disorder, and psychotic disorder not otherwise specified), (3) no head trauma, neurological or other medical disorder that could influence CNS functioning, and (4) IQ over 70.

The distribution of diagnoses in the present study was as follows: *N* = 160 (60%) with a diagnosis of schizophrenia, *N* = 72 (27%) schizophreniform disorder, and *N* = 33 (13%) schizoaffective disorder.

All participants gave their informed consent, and the Regional Committee for Medical Research Ethics and the Norwegian Data Inspectorate approved the study.

### Clinical Assessments

Sociodemographic history (age, gender, ethnicity, and education), smoking and alcohol use history, and psychiatric history, including duration of untreated psychosis (DUP), hospitalizations, and antipsychotic medications prescribed were obtained through structured interviews. Information on adherence to medication was gathered by the patients themselves, reporting on a scale from 0 to 100%, on how much of their medication they had taken during the past week. All patients were diagnosed with the Structural Clinical Interview for DSM-IV (SCID). Psychotic symptoms were assessed using The Positive and Negative Syndrome Scale (PANSS) (32). Item g12 from PANSS was used as a measure of insight into illness. Depression was assessed with the Calgary Depression Scale for Schizophrenia (CDSS) (33). The Global Assessment of Functioning scale (GAF), split version, was used to assess the general level of symptoms and functioning (34) while the Alcohol Use Disorders Identification Test (AUDIT) measured the extent of alcohol use (35).

### Measurement of Cannabis Use

Cannabis use was documented through self-reports and information from medical charts, as well as by screening cannabis metabolites in urine. In the present study, we thus had access to data concerning current cannabis use, lifetime cannabis use, and cannabis use disorders according to DSM IV criteria (Table 1). Current cannabis use was registered as positive (“yes”) if the patient had used cannabis within the last 2 weeks before the assessment. Lifetime use of cannabis was categorized into three groups: never used, used <10 times, or used 10 or more times. We chose to focus on lifetime use of cannabis since it provides a more robust indicator of the extent of cannabis use compared to potential variations in current use, and because consumption also below the threshold for a substance use diagnosis may influence clinical symptomatology in psychosis (36).

**Table 1:** Cannabis use in the FTP patients.

		***N* (%)**
Current cannabis use	No	190 (72)
	Yes	68 (26)
	Missing data	7 (3)
Lifetime cannabis use	Never	81 (31)
	<10 times	46 (17)
	≥10 times	109 (41)
	Missing data	29 (11)
DSM-IV diagnosis of cannabis use	No	147 (56)
	Abuse	14 (5)
	Dependence	30 (11)
	Missing data	74 (28)

*FTP, first treatment for psychosis; N, Number of patients; %, Percentage of patients; DSM-IV, Diagnostic and Statistical Manual of Mental Disorders 4th Edition*.

### Measurement of Attitudes Toward Medication

To measure patients' beliefs about their medication, we used the validated Norwegian version of the self-report form Beliefs about Medicines Questionnaire (BMQ) (Table 2) (28).

**Table 2:** Attitudes toward medication as measured by the Beliefs about Medicines Questionnaire (BMQ).

**BMQ subscales**	***N***	**Mean (SD)**	**Range**
“Specific Concerns” subscores	231	17.6 (4.7)	6–30
“Specific Necessity” subscores	230	15.8 (4.4)	5–25
“General Overuse” subscores	233	12.3 (2.7)	4–20
“General Harm” subscores	236	10.6 (2.4)	4–20

*BMQ, Beliefs about Medicines Questionnaire; N, Number of patients; SD, Standard deviation*.

The questionnaire comprises a specific and a general scale, each with two subscales. The first part, BMQ “Specific,” assess attitudes toward medicines prescribed for a specific illness focusing on the necessity of taking the medicines and concerns about taking them, divided into “Specific Necessity” and “Specific Concern.” “Specific Necessity” has five sections on a 1–5 scale Likert scale (1 = strongly disagree to 5 = strongly agree) covering the extent to which it is considered necessary to take the prescribed medicine (total score range 5–25), while “Specific Concern” consists of six sections covering the degree of concern regarding the use of currently prescribed medicines (total score range 6–30). High scores for “Specific Concerns” represent beliefs that the medications in question (here antipsychotics) have potentially negative consequences, while high scores for “Specific Necessity” indicate the patient's positive perception of the need to take their medications consistently.

BMQ “General” is the second part of the questionnaire, which covers more general beliefs about medicines as treatments, including the risk of overuse and potential for being harmful. The “General Harm” subscale consists of four sections covering notions that medication might be generally harmful, addictive or poisonous and thus should not be taken continuously (total score range 4–20). The “General Overuse” subscale consists of four sections including claims that doctors prescribe too many medications, and that medicines are used too much in general (total score range 4–20 points). High scores for “General Injury” and “General Overuse” indicate overall negative attitudes toward the use of medications as a treatment option.

### Statistical Analyses

All statistical analyses were performed using the Statistical Package for the Social Sciences (SPSS) version 25.0. Normality of the data was checked using histograms and QQ-plots. Group differences were analyzed using Chi-square tests for categorical variables and analyses of variance (ANOVA), with Tukey *post-hoc* test, for continuous variables. Significant *p*-value was preset to <0.05, two-sided.

For the primary analyses, we used a set of general linear model (GLM) analyses to investigate the relationship between lifetime cannabis use (independent variable) and the different BMQ subscales (dependent variables), controlling for differences in age and gender. Additionally, separate *post-hoc* GLM analyses, controlling for age and gender, were carried out to investigate whether significant group differences as found in the Chi-square and ANOVA tests for demographic factors, lifestyle, or illness-related factors confounded the relationships between lifetime use of cannabis and attitudes to medicine. Preliminary analyses were conducted to ensure assumptions of normality and linearity for the GLMs; no violations of the assumptions were found.

Finally, we conducted *post-hoc* power analyses using the statistical package G^*^Power (3.1.9.3 for Mac).

#### Missing Data

There was a lack of data for ~20% of the patients when combining lifetime cannabis use with BMQ scores. Initial analyses showed that there was no significant difference in sociodemographic factors (age, gender, ethnicity, and total years of education), lifestyle factors (daily tobacco smoking, alcohol use), illness-related factors (age of onset, DUP, total PANSS score, positive PANSS subscores, negative PANSS subscores, depressive symptoms, and global functioning), and use of antipsychotic medications between those who lacked data and those with full datasets (*p* > 0.05 for all).

## Results

### Sample Characteristics

Table 3 summarizes demographic and clinical characteristics with group comparisons. Patients with lifetime use of cannabis ≥10 times were more likely to be male, of younger age, have a lower age of onset of psychosis and consume alcohol and tobacco more frequently compared to those who have never used cannabis. Patients with lifetime use of cannabis ≥10 times were also more likely to lack insight into illness compared to patients with lifetime cannabis use <10 times, as measured by the PANSS g12 subscore. There were no significant group differences in ethnicity, education, DUP, psychotic symptoms, depressive symptoms, global functioning, current antipsychotic treatment, or self-reported adherence to medication between the three cannabis use groups.

**Table 3:** Comparison of lifetime cannabis use vs. demography, lifestyle, illness-related factors, and beliefs about medication in FTP patients.

	**Subgroups of lifetime cannabis use**	***N***	**Mean (SD)/%**	***F*/Chi**	***P*-value**	***Post-hoc* tests for significant group differences in lifetime cannabis use**
**Demography**						
Gender (male)	1. Never	43	28	16.3	<0.001	1 vs. 3. 2 vs. 3
	2. <10 times	24	16			
	3. ≥10 times	85	56			
Age (year)	1. Never	81	28.8 (8.7)	6.2	0.002	1 vs. 3
	2. <10 times	46	26.9 (9.2)			
	3. ≥10 times	109	25.0 (5.3)			
Ethnicity (Caucasian)	1. Never	59	32	1.4	0.51	NA
	2. <10 times	37	20			
	3. ≥10 times	88	48			
Education (total years)	1. Never	80	13.0 (3.0)	2.6	0.08	NA
	2. <10 times	46	12.3 (3.0)			
	3. ≥10 times	109	12.2 (2.0)			
Tobacco smoking (daily)	1. Never	25	18	48.7	<0.001	1 vs. 2. 1 vs. 3
	2. <10 times	32	22			
	3. ≥10 times	86	60			
Alcohol use (AUDIT total score)	1. Never	72	4.8 (5.5)	7.7	<0.001	1 vs. 3
	2. <10 times	41	7.1 (7.6)			
	3. ≥10 times	95	9.2 (8.1)			
**Illness-related factors**						
Age of onset of psychosis	1. Never	79	24.9 (8.6)	4.1	0.02	1 vs. 3
	2. <10 times	45	22.2 (8.2)			
	3. ≥10 times	108	21.9 (5.7)			
DUP (weeks)	1. Never	79	155.1 (269.9)	1.2	0.29	NA
	2. <10 times	46	169.7 (217.4)			
	3. ≥10 times	108	117.1 (164.9)			
Overall psychotic symptoms (PANSS total score)	1. Never	81	67.7 (16.5)	0.6	0.53	NA
	2. <10 times	46	64.9 (16.4)			
	3. ≥10 times	109	68.0 (15.9)			
Positive symptoms (PANSS positive subscores)	1. Never	81	16.6 (5.2)	1.25	0.29	NA
	2. <10 times	46	15.7 (5.7)			
	3. ≥10 times	109	17.2 (5.4)			
Negative symptoms (PANSS negative subscores)	1. Never	81	16.4 (6.7)	0.38	0.68	NA
	2. <10 times	46	15.7 (6.0)			
	3. ≥10 times	109	16.7 (6.2)			
Lack of insight (PANSS g12 subscore)	1. Never	81	2.7 (1.5)	6.14	0.003	2 vs. 3
	2. <10 times	46	2.2 (1.3)			
	3. ≥10 times	109	3.1 (1.4)			
Depressive symptoms (CDSS total score)	1. Never	73	6.6 (5.2)	1.36	0.26	NA
	2. <10 times	44	7.2 (5.2)			
	3. ≥10 times	106	5.8 (4.8)			
Global functioning (GAF-F subscore)	1. Never	81	42.1 (10.8)	0.75	0.47	NA
	2. <10 times	46	40.2 (9.6)			
	3. ≥10 times	109	40.3 (9.7)			
Global symptoms (GAF-S subscore)	1. Never	81	39.6 (7.8)	0.93	0.40	NA
	2. <10 times	46	40.8 (11.1)			
	3. ≥10 times	109	38.6 (9.8)			
Current antipsychotic use	1. Never	62	32	3.66	0.16	NA
	2. <10 times	38	20			
	3. ≥10 times	95	49			
Self-reported adherence to medication (%)	1. Never	59	86 (35)	0.52	0.60	NA
	2. <10 times	38	79 (41)			
	3. ≥10 times	91	81 (39)			
**Beliefs about medication (BMQ)**						
BMQ “Specific” total scores	1. Never	64	33.2 (5.5)	1.24	0.29	NA
	2. <10 times	39	34.6 (5.6)			
	3. ≥10 times	98	32.9 (6.1)			
BMQ “Specific Necessity” subscore	1. Never	65	15.3 (4.3)	1.28	0.28	NA
	2. <10 times	39	16.7 (4.8)			
	3. ≥10 times	99	15.6 (4.3)			
BMQ “Specific Concern” subscore	1. Never	65	17.9 (4.6)	0.42	0.66	NA
	2. <10 times	40	18.1 (4.5)			
	3. ≥10 times	98	17.4 (4.8)			
BMQ “General” total scores	1. Never	66	22.9 (4.9)	0.03	0.97	NA
	2. <10 times	40	23.2 (4.1)			
	3. ≥10 times	98	23.1 (4.3)			
BMQ “General Harm” subscore	1. Never	66	10.6 (2.6)	0.29	0.75	NA
	2. <10 times	41	10.9 (2.2)			
	3. ≥10 times	101	10.7 (2.4)			
BMQ “General Overuse” subscore	1. Never	68	12.4 (2.9)	0.10	0.91	NA
	2. <10 times	40	12.2 (2.6)			
	3. ≥10 times	98	12.4 (2.7)			

*FTP, first treatment for psychosis; N, number of patients; SD, standard deviation; %, percentage; F, F-test; Chi, Chi-square test; NA, not applicable; AUDIT, alcohol use disorder Identification Test; DUP, duration of untreated psychosis; PANSS, positive and negative syndrome scale; CDSS, calgary depression scale for Schizophrenia; GAF-F/S, the global assessment of Functioning scale (GAF, split version); BMQ, beliefs about medicines questionnaire information on adherence to medication was gathered by the patients themselves, reporting on a scale from 0 to 100%, on how much of their medication they had taken during the past week. P-values are obtained from one-way ANOVA and Chi-square test. For p-values that were significant in the ANOVA and Chi-square tests, group differences in lifetime cannabis use were examined by post-hoc Tukey*.

### The Relationship Between Lifetime Cannabis Use and Beliefs About Medication

After controlling for age and gender, patterns of lifetime cannabis use were not associated with negative perceptions about medication, neither specific beliefs about antipsychotic medication nor general beliefs about pharmacotherapy in general as measured by the BMQ (Table 4).

**Table 4:** General linear model (GLM) analyses examining the relationship between lifetime cannabis use and beliefs about medication (BMQ) in patients with FTP.

	***F***	**df**	***P*-values**	**Adjusted *R* squared for the whole model**
**BMQ “Specific” total scores**
Age	2.71	1	0.10	0.009
Gender	0.60	1	0.44	
Lifetime cannabis use	0.99	2	0.37	
Error		196		
**BMQ “Specific necessity” subscore**
Age	1.90	1	0.17	−0.006
Gender	0.00	1	0.97	
Lifetime cannabis use	0.23	2	0.80	
Error		198		
**BMQ “Specific Concern” subscore**
Age	0.39	1	0.53	−0.002
Gender	0.71	1	0.40	
Lifetime cannabis use	1.23	2	0.30	
Error		198		
**BMQ “General” total scores**
Age	0.25	1	0.62	−0.015
Gender	0.66	1	0.42	
Lifetime cannabis use	0.09	2	0.91	
Error		199		
**BMQ “General Harm” subscore**
Age	5.80	1	0.02	0.012
Gender	0.18	1	0.68	
Lifetime cannabis use	0.46	2	0.63	
Error		203		
**BMQ “General Overuse” subscore**
Age	1.66	1	0.20	−0.005
Gender	1.15	1	0.28	
Lifetime cannabis use	0.14	2	0.87	
Error		201		

*FTP, first treatment for psychosis; BMQ, beliefs about medicines questionnaire; F, F-test; df, degrees of freedom. Analyzed with GLM models while controlling for age and gender*.

Similarly, current use of cannabis or a DSM-IV diagnosis of cannabis use disorders was not associated with BMQ subscores (*p* > 0.05). Moreover, *post-hoc* GLM analyses based on lifetime use of cannabis controlling for age and gender, as well as separately controlling for the measures that were significant in the group comparisons tests (i.e., daily smoking, alcohol use, age of onset of psychosis, and lack of insight into illness), did not show any significant associations between lifetime use of cannabis and BMQ subscores (all *p* > 0.05).

*Post-hoc* power analyses showed that the statistical power in our study was 0.5 for the lifetime cannabis use against BMQ subscores; thus, medium power. The power analyses also showed that for achieving a strong power (>0.8) we would have needed a sample size of 408 subjects.

## Discussion

The main finding of the present study was that a history of cannabis use was not associated with negative beliefs about medications as measured with the BMQ in FTP. To our knowledge, this is the first study that has examined this relationship.

Previous studies of psychotic patients have indicated that negative beliefs about medications are major reasons for non-adherence to antipsychotic drug treatments (37, 38); and likewise, that cannabis use is a risk factor for poor adherence to drug treatment (23). While we initially hypothesized to find a link between cannabis use and negative believes about medication, the results of the present study do not support such a link. This could suggest that the relationship between cannabis use and poor adherence to medication may not necessarily be influenced or mediated by negative beliefs about drug treatment. Still, other reasons for a lack of association should also be considered. One important factor is type II error related to sample size. In the *post-hoc* power analyses, we found that the power was 0.5 indicating a medium sized power, and that a sample size of 408 would have been needed for achieving a strong power (>0.8). It is therefore possible that our sample size was insufficient for detecting weaker associations between lifetime cannabis use and negative beliefs about medication. Additionally, our data showed that in FTP with limited exposure to antipsychotic drug treatment the mean BMQ scores were in the middle of the scales (i.e., neither predominantly negative nor positive about medication) and the standard deviation between 2.4 and 4.7. The relatively neutral beliefs about drug treatment in this group of patients could have precluded us from observing a link. Moreover, it is reasonable to anticipate that current symptomatology may impact the acceptance of treatment. For instance, patients who have persecutory delusions may be disinclined to take prescribed medication. Cannabis use has also been associated with more severe positive/psychotic symptoms (10, 39). However, in the current study we found no significant differences in psychotic symptoms between the three lifetime cannabis groups. This may suggest that the participants were recruited in a stable phase of illness with low symptom levels that could influence any negative beliefs about medication.

We found that close to 70% of the participants had used cannabis at some point in their lives, with nearly 50% reporting frequent use (≥10 times) and 25 % meeting the DSM-IV diagnosis of cannabis use disorders, in accordance with previous findings (40, 41). Additionally, we found a significant gender effect with a higher prevalence of frequent cannabis use in males, in line with a recent Norwegian study of patients with FTP (42) and several prior studies (6, 43). Moreover, cannabis users were younger than those with no prior use (11, 16, 17), had higher alcohol consumption (44–46), were more often smokers (47), had a lower age at onset of psychosis (39, 48, 49), and showed poorer insight into illness (23); indicating that our sample, despite of the possible limitations described in the previous section, may be representative of FTP and that our findings could generalize outside of the current settings.

### Limitations

The sample consisted of psychiatric patients who had given informed consent to participate in a comprehensive research project, this might have caused a bias in the direction of more adherent patients in our sample. It is also possible that patients who are skeptical about doctors, medical treatment or research, as well as those with more pronounced delusions, may have said no to be included in the study. Our patients were relatively young and with limited antipsychotic experiences, this could have resulted in more neutral BMQ scores. Our results may therefore not necessarily be transferable to chronic patients. Also, we did not have information on whether the patients met the criteria for DSM-IV diagnosis of tobacco and/or alcohol abuse and dependence. Additionally, the study was medium powered and well-powered studies are needed before firm conclusions can be made.

### Strengths and Clinical Implications

To our knowledge, this is the first study in a psychosis cohort that has specifically examined a relationship between cannabis use and negative perceptions toward medication. The FTP sample was well-characterized, allowing us to examine group differences in demographic, lifestyle, illness, and treatment related factors.

Cannabis use and poor compliance to antipsychotic medication is a huge problem in patients with psychosis. Not only does it affect the lives of the patient in terms of poorer illness course and outcome, but it also effects their care givers and the society, e.g., readmissions to hospitals and extended stays in hospital wards. In order to device targeted approaches to address this problem of poor compliance, it is important to understand any additional factors that may contribute to poor drug adherence. The findings of the present study may therefore be of clinical relevance as they could point to other factors besides negative attitudes and believes about medication being important for poor adherence in cannabis users and that other aspects of cannabis use should be explored in future studies in order to improve treatment adherence in this patient group.

## Conclusion

The present study show that a history of cannabis use is not associated with negative perceptions toward medications among patients with FTP. This may be a characteristic of an FTP sample with limited positive and negative experiences with antipsychotic treatments, and illness development could change their attitudes. Future studies should examine this association in well-powered longitudinal studies with multiple time points and objective adherence measures to better understand the relationship between cannabis use, believes about medication, and adherence to drug treatment.

## Data Availability Statement

The datasets presented in this article are not readily available because sharing of data to external parties has not been approved by the Ethics Committee. Requests to access the datasets should be directed to PG, prig@norceresearch.no.

## Ethics Statement

The studies involving human participants were reviewed and approved by Regional Committee for Medical Research Ethics. The patients/participants provided their written informed consent to participate in this study.

## Author Contributions

SS designed the study, together with IM and VS. PG and SS analyzed the data and were responsible for interpretation of results together with IM and VS. PG drafted the first version of the manuscript together with IM and VS. OA, NS, TV, and ER contributed with data. All authors contributed to the writing of the manuscript and approved the final version.

## Funding

This study was supported by grants from the Research Council of Norway to NORMENT CoE (grant number 223273/F50, under the Centers of Excellence funding scheme), South-Eastern Norway Regional Health Authority (#2006233, #2006258, #2011085, #2014102, and #2015088), and Stiftelsen Kristian Gerhard Jebsen (SKGJ-MED-008). The funding bodies had no role in the analyses or writing of the manuscript, or the decision to submit this work for publication.

## Conflict of Interest

OA has received speaker's honorarium from Lundbeck and was a consultant for HealhLytix. The remaining authors declare that the research was conducted in the absence of any commercial or financial relationships that could be construed as a potential conflict of interest.

## Publisher's Note

All claims expressed in this article are solely those of the authors and do not necessarily represent those of their affiliated organizations, or those of the publisher, the editors and the reviewers. Any product that may be evaluated in this article, or claim that may be made by its manufacturer, is not guaranteed or endorsed by the publisher.
